# Examining suicide risk among people with schizophrenia, focusing on the role of anosognosia and the ethical considerations for future research directions

**DOI:** 10.3389/fpsyt.2025.1698101

**Published:** 2025-10-20

**Authors:** Carla Kotzé, Johannes Lodewikus Roos

**Affiliations:** Weskoppies Hospital, Gauteng Health and Department of Psychiatry, Faculty of Health Sciences, University of Pretoria, Pretoria, South Africa

**Keywords:** schizophrenia, suicide, risk factors, anosognosia, depression, ethics

## Abstract

This perspective paper discusses the primary risk factors consistently identified in systematic reviews of suicide among individuals with schizophrenia. Particular attention is given to the intricate relationship between anosognosia (lack of insight into one’s illness) and suicide risk in this population. This paper explores how anosognosia may influence other established risk factors and potentially contribute to suicidal behavior in patients with schizophrenia. By analyzing this complex interplay, this paper aims to provide a more comprehensive understanding of suicide risk in schizophrenia, potentially informing more effective prevention strategies and interventions for this vulnerable group. Future research in this area should carefully consider the ethical implications, including the potential impact of interventions on individuals with varying levels of insight into their condition. Researchers must prioritize participant safety and well-being while balancing the need to advance knowledge in this critical field.

## 1 Introduction

Suicide is a significant concern in individuals with schizophrenia, with an estimated lifetime risk of approximately 5%; however, predicting suicide in individuals with schizophrenia remains inaccurate and challenging (1). This perspective paper considers the major risk factors that have been repeatedly found in systematic reviews of the risk factors for suicide in patients with schizophrenia, specifically considering the complex interplay between anosognosia and suicide risk. Due to the limited improvement in our understanding and ability to prevent suicide in individuals with schizophrenia, this paper focuses on future areas of research, with specific ethical considerations for these research directions.

## 2 Systematic reviews of risk factors for suicide in individuals with schizophrenia

Hawton et al. conducted a systematic review in 2005, focusing on patients with schizophrenia and suicide as a reported outcome. They identified affective symptoms as a major risk factor. In assessing affective symptoms, distinguishing whether depression is an integral aspect of schizophrenia, a subsequent occurrence, or an independent disorder presents a challenge. Additional risk factors include suicidal ideation or actions, poor treatment adherence, and substance abuse. Their conclusions emphasized the need to address affective symptoms, improve treatment adherence, and maintain awareness of risks, especially after significant losses (2). Hor and Taylor identified overlapping risk factors in a 2010 systematic review of suicide and schizophrenia. Beyond the factors in **Table 1**, they found that insight was a suicide risk, to be considered later when addressing anosognosia. Depression and hopelessness, along with physical illnesses, were major risk factors. Protective factors include adequate treatment for schizophrenia and comorbidities (3).

**Table 1:** Summary of key suicide risk factors in patients with schizophrenia.

**Major:** Affective symptoms / Depression / Anxiety Prior suicide attempts Frequency of psychiatric hospitalizations
**Other risk factors:** Younger age Recent illness onset (especially during the first year) Recent admission Male sex Substance abuse Unemployment Higher IQ Feelings of worthlessness / hopelessness Poor treatment adherence

(2–6).

In 2014, a systematic review and clinical recommendations on suicide risk factors in individuals with schizophrenia were published. This review confirmed the high risk of suicide in the presence of affective symptoms, prior suicide attempts, and frequency of psychiatric hospitalizations (4). Cassidy et al. (5) conducted a systematic review and meta-analysis of the risk factors associated with suicidal ideation, attempts, and suicide in patients with schizophrenia. This review identified shorter illness length, younger age, and higher IQ as continuous risk factors, in addition to the other risk factors included in **Table 1** (5). Barbeito et al. (6) specifically investigated suicide risk in adolescents with psychotic disorders and in keeping with the other reviews highlighted that the period of greatest risk for suicide attempts occurs very early in the disorder. Specifically, before their first hospital admission or within the first month of hospitalization. Most attempts happened while patients were being treated as outpatients. These findings align with research on first-episode psychosis in general, which identifies the period before and immediately after initial treatment as the highest risk (6).

**Table 1** summarizes the key suicide risk factors in patients with schizophrenia identified in these systematic reviews.

A meta-analysis concluded that the ability to use risk factors to predict suicidal thoughts and behaviors has not advanced over the past five decades (7). The recommendations for suicide prevention include the following:

- Close monitoring for inpatients with schizophrenia, with a history of suicide attempts, depressive symptoms, or frequent psychiatric hospitalizations,
- Conduct immediate suicide risk assessments for all newly admitted patients.
- Maintain close supervision following hospital discharge.
- Take into account any family history of suicide.
- Provide closer monitoring for younger patients in the early stages of illness.
- Examine potential comorbid substance abuse.
- Screen all patients with schizophrenia for depression and feelings of hopelessness.
- Inquire about suicidal ideation during each clinical consultation.
- Confirm compliance with prescribed treatments.
- Assess levels of impulsivity.
- Ensure inpatient facilities are aware of and can implement appropriate suicide prevention measures.
- Educate caregivers and family members about potential suicide methods.
- Thoroughly investigate the circumstances surrounding previous suicide attempts.
- Admit patients with schizophrenia experiencing acute episodes with suicidal ideation.

These reviews noted a lack of standardization in assessing suicidality in patients with schizophrenia and suggested longitudinal studies to explore the mechanisms linking identified variables to suicidality, as well as studies incorporating machine learning techniques to objectively predict suicide risk at an individual level in patients with schizophrenia (2, 4, 5).

## 3 Assessment of suicide risk in individuals with schizophrenia

Suicide among individuals with schizophrenia remains a complex clinical challenge. The assessment of suicide risk in this group is influenced by demographic, clinical, psychological, social, cultural, and environmental factors. Mental health professionals working with individuals with schizophrenia may have experienced instances of suicide despite risk assessment efforts. Suicide risk assessments often yield false-positive results. The emphasis in managing suicide risk is shifting from suicide risk assessment (SRA) to suicide risk formulation (SRF) using information from the SRA process. Suicide risk levels guide treatment and management strategies (8, 9). The SRF process considers four key areas, as summarized in **Table 2**.

**Table 2:** Four key areas of suicide risk formulation.

Key area	Strategy
Risk status	Assess demographic factors and other characteristics that place the patient in a higher risk group
Risk state	Evaluate the patient’s current risk level by performing regular assessments to track changes in risk over time and identify periods of elevated risk
Available resources	Identify support systems accessible to the patient and help strengthen the support network and provide information on crisis services
Foreseeable events	Anticipate potential triggers or stressors that may exacerbate risk and work with patient to prepare for upcoming events or transitions (e.g. discharge from hospital)

(8, 9).

Multiple risk factors should be assessed to understand the individual risks. The context should be considered without overemphasizing the diagnosis. Mental health practitioners should evaluate patients’ clinical context, history, and circumstances. This is crucial because some patients may not disclose their suicide plans. However, this should not prevent inquiries regarding the means of suicide. Suicide prevention can be enhanced by assessing multiple risk factors using the SRF. This approach provides an understanding of the risks associated with schizophrenia. SRF should be considered when developing research protocols for suicide risk studies in individuals with schizophrenia, considering a broader context instead of emphasizing diagnosis (8, 9).

## 4 Anosognosia and its relationship to suicide in patients with schizophrenia

Poor insight is considered a symptom of schizophrenia related to underlying brain abnormalities rather than a coping strategy, and has a complex relationship with outcomes. Lack of insight or anosognosia is one of the most prevalent symptoms of schizophrenia (10). It commonly predicts medication nonadherence and leads to inaccuracies in patient self-assessment, thereby impacting functional outcomes. Research has shown a strong correlation between executive dysfunction, verbal processing deficits, and lack of illness awareness in patients with schizophrenia. Abnormalities in the frontal lobe and prefrontal cortex have been observed in patients with poor awareness. This indicates that poor insight cannot be explained as denial, and anosognosia is an important risk factor for suicidality in patients with schizophrenia. However, this relationship is only relevant with feelings of hopelessness and demoralization (11).

A meta-analysis of brain areas involved with clinical and cognitive insight in psychotic disorders found that clinical insight relates to diffuse abnormalities across the brain, while cognitive insight is mainly associated with the ventrolateral prefrontal cortex and hippocampal areas. Illness insight is a distinct symptom domain that does not entirely overlap with the cognitive domain of illness (12). According to Carrara (13), being diagnosed with schizophrenia alters how individuals perceive and understand their environment. Schizophrenia disrupts cognitive function and emotional stability, leading to changes in awareness and a diminished capacity to respond to stimuli. The clarity of conscious thought is altered by schizophrenia and affects a person’s ability to make informed choices that reflect their true intentions (13).

Examination of illness unawareness revealed that introspective accuracy and bias offer a broader understanding of anosognosia in schizophrenia. Introspective accuracy refers to the ability to accurately assess the domains of experience and functioning. Introspective bias refers to the tendency of people to inaccurately understand their thoughts or feelings, which affects their responses. The patterns of mis-estimation in these patients include overestimation of abilities and underestimation of task difficulties. This may contribute to difficulties with treatment adherence, increased symptoms, greater disability in everyday living, reduced response to cognitive interventions, and a need for increased intensity of interventions (14).

Melle and Barret (1) examined the role of insight in suicidal behavior among patients with a first episode of schizophrenia. They found that better illness insight could increase the suicide risk in some patients. This relationship is complex and may be influenced by stigma and patients’ beliefs about psychotic disorders (1). Berardelli et al. (15) explored the connection between good insight, low self-esteem, and diminished quality of life among individuals with schizophrenia. Their review identified hopelessness as a key predictor of current and lifetime suicidality among patients with depression, independent of depression. They found that patients with heightened insight were more likely to feel demoralized due to self-stigma, suggesting that insight may influence suicide risk through demoralization rather than directly affecting suicidal behavior. This challenges the view of insight as solely beneficial and highlights the need for comprehensive suicide prevention strategies (15). Tayfur et al. (16) reported similar findings, where better insight and depression predicted suicidality at 6 and 12 months during first-episode of psychosis. This demonstrates the ‘insight paradox, ‘ where better insight increases suicidality, mediated by depression. (16). The direction of causality between greater insight, lower mood, and suicidality remains unclear and requires further investigation (17).

Due to the insight paradox, psychoeducational interventions and cognitive behavioral therapy may pose risks, as increased awareness can temporarily increase suicidal thoughts. Increasing patient awareness of illness may harm quality of life if not paired with interventions that help patients understand their experiences and maintain hope. Measures addressing psychological distress and reducing internalized stigma should accompany such psychoeducational interventions. This highlights the need to monitor suicidal ideation, depression, and insight during treatment, especially during inpatient-to-outpatient transitions (1, 18).

A link between insight and suicide risk has also been challenged by some researchers, who have shown that it is not a significant contributing risk factor. Merve et al. (19) found that depression and internalized stigma, where a person endorses negative societal stereotypes about a psychiatric illness, were more significant risks for suicide in patients with schizophrenia. The findings underscore the complexity of managing suicide risk in schizophrenia and the need for comprehensive personalized evaluation and targeted interventions in the clinical management of patients with schizophrenia to reduce suicide risk (20). Insight is dynamic, and any changes in the level of insight might serve as a warning sign for increased suicide risk (21).

## 5 Future research directions and ethical considerations for enhancing our understanding of suicide in individuals with schizophrenia

### 5.1 Neurocognitive profile and suicide risk in individuals with schizophrenia

Research on social cognition, general cognition, and suicide risk has shown that individuals with psychiatric disorders exhibit increased sensitivity to social-emotional cues and diminished attentional control and learning abilities. An inefficient capacity to manage perceived social stress contributes to suicidal thoughts and behaviors (22). Social cognition is the capacity to observe, identify, and reason about one’s own emotions and those of others. This capacity is more impaired in individuals who attempt suicide than in those who have suicidal ideation (23).

A systematic review by Sanchez-Gutiérre et al. (24) concluded that patient with first-episode psychosis and suicidal behaviors exhibit more varied and poorer neuropsychological functioning compared to first episode psychosis patients without suicidal behavior, particularly in the early stages of the illness. Key areas of deficit associated with a higher risk of suicidal behavior include processing speed, visual memory, and psychomotor skills like motor dexterity and speed. However, these associations tend to diminish over time (24).

Additional research is needed to elucidate the significance of these social and nonsocial cognitive factors. Future studies should include everyday measures of social cognition, different conceptualizations of suicidal ideation, and theory of mind with both positive and negative affects (22). Developing a comprehensive neurocognitive profile of the factors contributing to suicide in individuals with schizophrenia is essential.

### 5.2 Suicide capability

A comprehensive review of suicide capabilities identified cognitive function as a contributing factor. Individuals who attempted suicide exhibited significantly more impairment in cognitive functioning than those with suicidal ideation. Suicidal capability refers to the progression from suicidal ideation to suicide attempts or completion (25). The three primary components of suicide capability are as follows:

1. Acquired aspects – reduced fear of death, increased pain tolerance, abuse in childhood.
2. Dispositional factors – genetics, temperament, personality traits.
3. Practical aspects – knowledge of and access to lethal means (25).

Most studies on suicide capability have focused on a single factor within these components. Future research should examine the interplay between multiple factors to better understand the phenomenon of suicide capability, as discussed in the algorithm section of this paper.

### 5.3 Adverse childhood experiences and suicidal behavior in individuals with schizophrenia

A meta-analysis by Baldini et al. (26) showed that exposure to adverse childhood events significantly increases suicidal behavior in people with schizophrenia spectrum disorders. One-third of individuals with schizophrenia spectrum disorders experience adverse childhood events associated with a severe disease course and treatment resistance (26). Researchers struggle to obtain this information due to the retrospective nature of studies and subject sensitivity. Screening for adverse childhood experiences should be incorporated into routine data collection in suicide risk studies involving individuals with schizophrenia. Characterizing adverse childhood experiences in individuals with schizophrenia spectrum disorders is crucial for improving suicide risk assessment (26).

Additional efforts are required to obtain sensitive information. Alternative sources may be required when an individual dies by suicide. Ensuring sensitivity to ethical considerations is crucial when collecting this data. Research on adverse childhood experiences and suicide risk in individuals with schizophrenia involves complex ethical considerations. These studies require a thoughtful approach to research design and implementation. Gathering data on adverse childhood experiences requires participants to share potentially traumatic events, risking emotional distress (26). Researchers must ensure that participants understand the questions and their potential emotional impact. A balance between comprehensive data collection and minimization of psychological harm is essential. This requires trauma-informed interview techniques, mental health support during data collection, and allowing participants to withdraw without consequences (26–28).

Studying adverse childhood experiences in suicide cases presents several ethical challenges. Researchers must balance the benefits of research with the protection of the privacy of the deceased and their families. Accessing records or interviewing family members requires careful consideration of consent. Researchers must consider the impact on surviving family members when discussing childhood trauma and suicide (28–30). Another concern is the risk of stigmatization or oversimplification of the relationship between adverse childhood events and suicide. While this research could provide insights into suicide prevention, there is a danger of implying a direct causal link that may not capture the complexity of individual cases. Researchers must frame their findings to avoid reinforcing harmful stereotypes or blaming families (31). Furthermore, methodological challenges exist in retrospectively assessing adverse childhood experiences in deceased individuals, potentially leading to incomplete data. Addressing these ethical dilemmas requires robust review processes, clear protocols for data handling, and dialogue with stakeholders in the mental health and suicide prevention communities (32, 33).

### 5.4 Use of technology and transitioning from risk factors to risk algorithms

Franklin et al. (7) suggested shifting from identifying suicide risk factors to developing risk algorithms, aligning with the previously discussed SRF (7, 8). Machine learning approaches autonomously determine optimal predictive algorithms and can model complex predictor relationships, yielding accurate models for forecasting suicidal thoughts and behaviors (7). Risk algorithms must incorporate established suicide risk factors for patients with schizophrenia in an intricate and reproducible manner. Machine learning methods excel in analyzing complex relationships among multiple predictors (34). Machine learning algorithms are essential for the accurate prediction of suicidal thoughts and behaviors. Technology enables frequent assessment of risk factors on a minute or hourly basis. While most studies have focused on single factors, future research should explore the algorithmic interactions between multiple factors. Cognitive and affective tasks can now be adapted for mobile applications. Advanced mobile technologies enable the swift and cost-effective recruitment of thousands of at-risk individuals worldwide (35).

If we consider future research aspects of suicide in schizophrenia, we must always keep in mind that completed suicide is a complex biopsychosocial phenomenon. This is unlikely to be explained by any single factor, including depression or anosognosia. Causative or contributing factors in the suicide puzzle can evolve over time, and artificial intelligence (AI) would be best situated to evaluate these complex evolving factors (17). If we look at future research aspects of suicide in patients with schizophrenia, the application of advanced technologies such as AI and wearable devices shows promise in preventing suicide among patients with psychiatric disorders, including those diagnosed with schizophrenia. For example, machine learning algorithms can analyze patterns in electronic health records, social media activity, and physiological data from wearables to predict suicidal behavior. Although it may not currently be ready for clinical implementation, this predictive capability could enable timely intervention, potentially saving lives (36–39).

However, the implementation of these technologies raises ethical questions regarding privacy, data security and informed consent. AI-driven tools often require extensive personal data, posing a risk of misuse or confidentiality breaches. Consent must be explicit, with participants understanding how their information will be stored. In addition, the potential for AI bias must be considered, as an algorithm may inaccurately assess suicide risk, leading to false positives or negatives that affect patient care. Ultimately, the ethical use of these technologies requires transparency, robust data protection measures, and careful supervision (40, 41). For suicide prediction tools to be effective and clinically relevant, they must evolve to be dynamic, explainable, and capable of providing actionable insights that inform real-time, personalized interventions. The development of these systems must involve co-designing with individuals who have lived experience, clinicians, and healthcare services to ensure they are effective and patient-centered (42).

## 6 Conclusion

This perspective paper examined the complex relationship between suicide risk and anosognosia in individuals with schizophrenia and the ethical considerations for future research on suicide risks in this patient population. Evidence suggests that poor insight is a significant risk factor for suicide in this population, but its effects are mediated by feelings of hopelessness and demoralization. Future research should focus on developing more comprehensive risk algorithms with SRF that incorporate multiple factors, including neurocognitive profiles, adverse childhood experiences and dynamic changes in insight levels. The use of advanced technologies, such as machine learning and wearable devices, shows promise for improving suicide risk prediction but raises important ethical considerations regarding privacy and consent. Moving forward, a holistic approach that balances scientific inquiry with ethical responsibility is crucial for enhancing our understanding of suicide risk in schizophrenia and developing more effective prevention strategies. By carefully addressing these complex issues, researchers can work towards reducing suicide rates and improving outcomes for this vulnerable population.

## Data Availability

The original contributions presented in the study are included in the article/supplementary material. Further inquiries can be directed to the corresponding author.

## References

[1] Melle I Barret AE. Insight and suicidal behavior in first-episode schizophrenia. Expert Rev Neurother. (2012) 12:353–9. doi: 10.1586/ern.11.191, PMID: 22364334

[2] Hawton K Hawton K Sinclair J Sinclair J Haw C Haw C. Schizophrenia and suicide: systematic review of risk factors. Br J Psychiatry. (2005) 187:9–20. doi: 10.1192/bjp.187.1.9, PMID: 15994566

[3] Hor K Taylor M. Suicide and schizophrenia: a systematic review of rates and risk factors. J Psychopharmacol. (2010) 24:81–90. doi: 10.1177/1359786810385490, PMID: 20923923 PMC2951591

[4] Popovic D Goikolea JM Vieta E Crespo JM González-Pinto A Benabarre A. Risk factors for suicide in schizophrenia: systematic review and clinical recommendations. Acta Psychiatrica Scand. (2014) 130:418–26. doi: 10.1111/acps.12332, PMID: 25230813

[5] Cassidy RM Yang F Kapczinski F Passos IC. Risk factors for suicidality in patients with schizophrenia: A systematic review, meta-analysis, and meta-regression of 96 studies. Schizophr Bull. (2017) 44:787–97. doi: 10.1093/schbul/sbx131, PMID: 29036388 PMC6007264

[6] Barbeito S Vega P Sánchez-Gutiérrez T Becerra JA González-Pinto A Calvo A. A systematic review of suicide and suicide attempts in adolescents with psychotic disorders. Schizophr Res. (2021) 235:80–90. doi: 10.1016/j.schres.2021.07.029, PMID: 34332428

[7] Franklin JC Ribeiro JD Fox KR Bentley KH Kleiman EM Huang X. Risk factors for suicidal thoughts and behaviors: A meta-analysis of 50 years of research. Psychol Bull. (2017) 143:187–232. doi: 10.1037/bul0000084, PMID: 27841450

[8] Berardelli I Erbuto D Sarubbi S Pompili M Lester D Rogante E. The importance of suicide risk formulation in schizophrenia. Front Psychiatry. (2021) 12. doi: 10.3389/fpsyt.2021.779684, PMID: 34975579 PMC8716825

[9] Shamabadi A Ahmadzade A Pirahesh K Hasanzadeh A Asadigandomani H. Suicidality risk after using cannabis and cannabinoids: An umbrella review. Dialog Clin Neurosci. (2023) 25:50–63. doi: 10.1080/19585969.2023.2231466, PMID: 37427882 PMC10334849

[10] Amador X. Denial of anosognosia in schizophrenia. Schizophr Res. (2023) 252:242–3. doi: 10.1016/j.schres.2023.01.009, PMID: 36682314

[11] Eneman M Vanhee L Tang E Sabbe B Corveleyn J Luyten P. The role of demoralization in the relationship between insight and suicidality in schizophrenia. Neurolo Psychiatry Brain Res. (2020) 38:12–5. doi: 10.1016/j.npbr.2020.08.002

[12] Pijnenborg GHM Larabi DI Xu P Hasson-Ohayon I De Vos AE Ćurčić-Blake B. Brain areas associated with clinical and cognitive insight in psychotic disorders: A systematic review and meta-analysis. Neurosci Biobehav Rev. (2020) 116:301–36. doi: 10.1016/j.neubiorev.2020.06.022, PMID: 32569706

[13] Carrara A. A neuroethical approach to human life, identity, and liberty of schizophrenic patients. CNS Spectrums. (2025) 30:e4. doi: 10.1017/S1092852924000506, PMID: 39665237 PMC13064752

[14] Rose B Harvey PD. Anosognosia in schizophrenia. CNS Spectrums. (2024) 30:1–21. doi: 10.1017/S1092852924002323, PMID: 39721947 PMC13064738

[15] Berardelli I Sarubbi S Rogante E Hawkins M Cocco G Erbuto D. The role of demoralization and hopelessness in suicide risk in schizophrenia: A review of the literature. Medicina. (2019) 55:200. doi: 10.3390/medicina55050200, PMID: 31126145 PMC6571661

[16] Tayfur SN Song Z Li F Hazan H Gibbs-Dean T Purushothaman D. Insight and suicidality in first-episode psychosis: The mediating role of depression. Schizophr Res. (2025) 275:189–95. doi: 10.1016/j.schres.2024.12.013, PMID: 39754935 PMC12317406

[17] Lopez-Morinigo J-D David AS. Is too much insight bad for you? Br J Psychiatry. (2024) 225:454–7. doi: 10.1192/bjp.2024.173, PMID: 39422143

[18] Davis BJ Lysaker PH Salyers MP Minor KS. The insight paradox in schizophrenia: A meta-analysis of the relationship between clinical insight and quality of life. Schizophr Res. (2020) 223:9–17. doi: 10.1016/j.schres.2020.07.017, PMID: 32763114

[19] Bat Tonkuş M Çalışkan BB Alagöz E Schmaal L. The relationship between suicide and hopelessness in young adults aged 18-30: A systematic review. J Psy Nurs. (2022) 13:253–62.

[20] Bilgin Koçak M Rıfat Şahin A Güz H Böke Ö Sarısoy G Karabekiroğlu A. The relationship between suicide attempts and ideation with depression, insight, and internalized stigmatization in schizophrenia. Alpha Psychiatry. (2021) 23:18–25. doi: 10.5152/alphapsychiatry.2021.21216, PMID: 36425246 PMC9674101

[21] Ayesa-Arriola R Terán JMP Moríñigo JDL Rivero MC Setién-Suero E Al-Halabi S. The dynamic relationship between insight and suicidal behavior in first episode psychosis patients over 3-year follow-up. Eur Neuropsychopharmacol. (2018) 28:1161–72. doi: 10.1016/j.euroneuro.2018.05.005, PMID: 30097249

[22] Dickhoff J Opmeer EM Heering HD Bruggeman R Van Amelsvoort T Bartels-Velthuis AA. Relationship between social cognition, general cognition, and risk for suicide in individuals with a psychotic disorder. Schizophr Res. (2021) 231:227–36. doi: 10.1016/j.schres.2021.02.024, PMID: 34000502

[23] Comparelli A Bargagna P Forcina F Corigliano V Lamis D Nardella A. Building a neurocognitive profile of suicidal risk in severe mental disorders. BMC Psychiatry. (2022) 22:1–8. doi: 10.1186/s12888-022-04240-3, PMID: 36162995 PMC9511976

[24] Sánchez-Gutiérrez T Barbeito S Gómez-Juncal R Rodríguez-Ortega E Becerra-García JA Calvo A. Neuropsychological functioning and suicidal behaviours in patients with first-episode psychosis: A systematic review. Acta Psychiatr Scand. (2022) 146:515–28. doi: 10.1111/acps.13501, PMID: 36153777

[25] Bayliss LT Christensen S Lamont-Mills A Du Plessis C. Suicide capability within the ideation-to-action framework: A systematic scoping review. PloS One. (2022) 17:e0276070. doi: 10.1371/journal.pone.0276070, PMID: 36301944 PMC9612581

[26] Baldini V Stefano RD Rindi LV Ahmed AO Koola MM Solmi M. Association between adverse childhood experiences and suicidal behavior in schizophrenia spectrum disorders: A systematic review and meta-analysis. Psychiatry Res. (2023) 329:115488. doi: 10.1016/j.psychres.2023.115488, PMID: 37769371

[27] Sasaki N Watanabe K Kanamori Y Tabuchi T Fujiwara T Nishi D. Effects of expanded adverse childhood experiences including school bullying, childhood poverty, and natural disasters on mental health in adulthood. Sci Rep. (2024) 14:12015. doi: 10.1038/s41598-024-62634-7, PMID: 38797740 PMC11128446

[28] Sachs-Ericsson NJ Rushing NC Stanley IH Sheffler J. In my end is my beginning: developmental trajectories of adverse childhood experiences to late-life suicide. Aging Ment Health. (2016) 20:139–65. doi: 10.1080/13607863.2015.1063107, PMID: 26264208

[29] Navalta CP Mcgee L Underwood J. Adverse childhood experiences, brain development, and mental health: A call for neurocounseling. J Ment Health Couns. (2018) 40:266–78. doi: 10.17744/mehc.40.3.07

[30] Fuller-Thomson E Dhrodia R Brennenstuhl S Baird SL. The association between adverse childhood experiences (ACEs) and suicide attempts in a population-based study. Child: Care Health Dev. (2016) 42:725–34. doi: 10.1111/cch.12351, PMID: 27280449

[31] Agteren J Iasiello M. Advancing our understanding of mental wellbeing and mental health: The call to embrace complexity over simplification. Aust Psychol. (2020) 55:307–16. doi: 10.1111/ap.12440

[32] Larkin H Felitti VJ Anda RF. Social work and adverse childhood experiences research: implications for practice and health policy. Soc Work Public Health. (2013) 29:1–16. doi: 10.1080/19371918.2011.619433, PMID: 24188292

[33] Panagou C Macbeth A. Deconstructing pathways to resilience: A systematic review of associations between psychosocial mechanisms and transdiagnostic adult mental health outcomes in the context of adverse childhood experiences. Clin Psychol Psychother. (2022) 29:1626–54. doi: 10.1002/cpp.2732, PMID: 35266603 PMC9790303

[34] Pompili M Krausz M Girardi P Saarinen PI Tatarelli R Modestin J. Suicide risk in schizophrenia: learning from the past to change the future. Ann Gen Psychiatry. (2007) 6:1–22. doi: 10.1186/1744-859X-6-10, PMID: 17367524 PMC1845151

[35] Correll CU Taipale H Tiihonen J Smith N Bitter I Solmi M. Mortality in people with schizophrenia: a systematic review and meta-analysis of relative risk and aggravating or attenuating factors. World Psychiatry. (2022) 21:248–71. doi: 10.1002/wps.20994, PMID: 35524619 PMC9077617

[36] Boudreaux ED Wang B Agu E Davis-Martin RE Semeter J Simon G. Applying machine learning approaches to suicide prediction using healthcare data: overview and future directions. Front Psychiatry. (2021) 12. doi: 10.3389/fpsyt.2021.707916, PMID: 34413800 PMC8369059

[37] Chen Q Faraone SV Zhang-James Y Lichtenstein P Larsson H D’onofrio BM. Predicting suicide attempt or suicide death following a visit to psychiatric specialty care: A machine learning study using Swedish national registry data. PloS Med. (2020) 17:e1003416. doi: 10.1371/journal.pmed.1003416, PMID: 33156863 PMC7647056

[38] Parghi N Galynker I Chennapragada L Lok B Newkirk S Ahmedani B. Assessing the predictive ability of the Suicide Crisis Inventory for near-term suicidal behavior using machine learning approaches. Int J Methods Psychiatr Res. (2020) 30:1–12. doi: 10.1002/mpr.1863, PMID: 33166430 PMC7992291

[39] Lejeune A Le Glaz A Perron PA Sebti J Baca-Garcia E Walter M. Artificial intelligence and suicide prevention: a systematic review. Eur Psychiatry. (2022) 65:1–22. doi: 10.1192/j.eurpsy.2022.8, PMID: 35166203 PMC8988272

[40] Goktas P Grzybowski A. Shaping the future of healthcare: ethical clinical challenges and pathways to trustworthy AI. J Clin Med. (2025) 14:1605. doi: 10.3390/jcm14051605, PMID: 40095575 PMC11900311

[41] Saeidnia HR Ghiasi N Lund B Hashemi Fotami SG. Ethical considerations in artificial intelligence interventions for mental health and well-being: ensuring responsible implementation and impact. Soc Sci. (2024) 13:381. doi: 10.3390/socsci13070381

[42] Nguyen J Dwyer DB Nelson B Schmaal L. From static to dynamic prediction of suicide risk. JAMA Psychiatry. (2025). doi: 10.1001/jamapsychiatry.2025.2426, PMID: 40928793

